# A qualitative study of informal caregiver perceptions of the benefits of an early dementia diagnosis

**DOI:** 10.1186/s12913-024-10957-6

**Published:** 2024-04-24

**Authors:** Elyse Couch, Melissa Co, Christopher P. Albertyn, Matthew Prina, Vanessa Lawrence

**Affiliations:** 1 Department of Health Services and Population Research, King’s College London, London, UK; 2 Department of Health Services, Policy and Practice, Brown University School of Public Health, Providence, USA; 3 Department of Old Age Psychiatry, King’s College London, London, UK; 4 Population Health Sciences Institute, Newcastle University, Newcastle, UK

**Keywords:** Dementia, Mild cognitive impairment, Early diagnosis, Caregivers, Qualitative research

## Abstract

**Background:**

Current and former dementia policies in the United Kingdom (UK) recommend diagnosing dementia early, or as close to the onset of symptoms as possible. Informal caregivers play an important role in initiating the diagnostic process and providing support to people living with dementia. Therefore, this study aimed to explore caregiver perceptions of the benefits of an early diagnosis.

**Methods:**

We conducted semi-structured interviews with 12 current and former informal caregivers to people with dementia in the UK in 2020. We analysed the interviews using thematic analysis.

**Results:**

Benefits of an early diagnosis included: (1) protecting the person with dementia from financial or physical harm, (2) timely decision-making, and (3) access to services and treatments following a diagnosis. We identified three conditions necessary for the benefits of an early diagnosis to be felt: (1) adequate prognostic information, (2) someone to advocate on behalf of the person with dementia, and (3) a willingness to seek and accept the diagnosis.

**Conclusions:**

In this study, we identified how diagnosing dementia close to the onset of symptoms could be beneficial and the conditions necessary for these benefits to be felt. The findings highlight the importance of an early diagnosis for enabling people with dementia and caregivers to make practical arrangements and to access services. Further research is needed to build on the findings of this study by exploring the perspectives of people with dementia and by including a larger, more diverse sample of caregivers.

**Supplementary Information:**

The online version contains supplementary material available at 10.1186/s12913-024-10957-6.

## Introduction

It is estimated that there are 944,000 people with dementia in the United Kingdom (UK) [1] however, only two-thirds have received a formal diagnosis [2]. Previous and current dementia policies in the UK recommend not only increasing diagnosis rates but also identifying dementia as early as possible [3, 4]. An early diagnosis can be defined as a diagnosis made at the onset of symptoms [5]. In practice, it can be challenging to make an early diagnosis as dementia is characterised by a gradual onset of symptoms, which can be difficult to distinguish from typical age-related changes in cognition, particularly during the early stages of memory loss. Patients experiencing cognitive impairment that does not significantly impact their daily functioning may be diagnosed with mild cognitive impairment (MCI) [6]. MCI is a significant predictor of future dementia, with approximately 7% of people with MCI developing dementia annually [7, 8]. However, MCI is a heterogeneous clinical concept, with many underlying etiologies, and not all people with MCI have or will have dementia [9]. Despite these concerns, MCI could be a potentially helpful diagnosis for identifying those at greatest risk of developing dementia and intervening early [10]. It is hoped that by diagnosing dementia early in the disease trajectory, people with dementia and their caregivers will be able to engage with services and treatments to live well for longer. However, there is very little primary evidence exploring the relationship between an early diagnosis and subsequent outcomes or experiences.

Two systematic reviews have attempted to systematically synthesize the evidence supporting the proposed benefits of an early diagnosis [11, 12]. The World Alzheimer’s Report in 2011 reviewed studies assessing the relationship between the stage of the disease at the time of diagnosis and subsequent outcomes including the rate of cognitive or functional decline, mortality rates, the timing of care or nursing home admission, quality of life and wellbeing for the person with dementia and caregiver, caregiver burden, and healthcare utilisation and associated costs. They identified three studies, all with moderate risk of bias. One study found a diagnosis close to the onset of symptoms was associated with a reduced risk of mortality. Two studies examined the relationship between the severity of symptoms at the time of diagnosis and rates of cognitive decline, neither of which found significant effects [12]. Furthermore, when the authors reviewed statements in published papers proposing the benefits of early diagnosis, they found the assertions to be lacking in empirical support, stating: “Many were unreferenced, and where references were provided these were generally to other papers making similar, non-evidence-based assertions. These statements should therefore be considered, at best, to represent expert opinion” [12]. A later review conducted in 2016, similarly concluded that there is a paucity of research focused on benefits to people living with dementia or caregivers, and many of the proposed benefits are based on modelling studies rather than patient data [11].

Many people with dementia are supported by informal caregivers. It has been estimated that 75% of people with dementia are supported by informal caregivers [13]. Caregivers play an important role in initiating the diagnostic process and organising the care for the person with dementia. Caregivers for people with dementia are at risk of high levels of burden and have been described as “invisible second patients” [14]. In addition to benefiting people with dementia, there is a hope that diagnosing dementia early would also lead to positive outcomes for caregivers. A survey of dementia caregivers from 5 European countries found that 47% of participants would have preferred to receive the diagnosis earlier [15]. A dementia diagnosis can confirm caregivers’ suspicions or lead to feelings of relief [16]. Additionally, an early diagnosis could enable caregivers to adapt to their new role as a caregiver and prepare for the future [16]. For example, a study of US caregivers found early use of home and day care services was associated with delayed institutionalisation [17]. Furthermore, early adaptation to the caregiver role may reduce the risk of later negative psychological outcomes [18]. On the other hand, there is a concern that an early diagnosis may label relatives of people with dementia as caregivers prematurely [19]. In this study, we conducted a qualitative analysis of semi-structured interviews to explore caregiver perspectives on the benefits of an early diagnosis (or a diagnosis close to the onset of symptoms). We were specifically interested in understanding what benefits caregivers perceived to be associated with an early diagnosis and which conditions were necessary for an early diagnosis to be beneficial. These findings could be used to inform future initiatives to increase early diagnosis rates and improve outcomes for caregivers and people with dementia.

## Methods

We conducted a reflexive thematic analysis of semi-structured interviews. Ethical approval was granted by the National Health Service (NHS) Health Research Authority and Health and Care Research Wales Research Ethics Committee (Reference number: 19/WA/0210). This study was part of a larger mixed-methods PhD project examining the benefits of an early diagnosis.

### Participants

Participants were included if they were a current or former informal caregiver for a person with dementia or MCI, over the age of 18, and able to consent to participate in the interview. In this study, an informal caregiver was defined as someone providing care to a person with dementia with whom they typically have a personal relationship, and don’t receive payment. An informal caregiver could be a family member, friend, or neighbour. Paid or formal caregivers, persons under the age of 18, or persons who were not able to give informed consent were not included.

### Recruitment

Participants were identified through two recruitment channels, Join Dementia Research (JDR) and support groups in South London, UK. JDR is an online self-registration service that enables volunteers with memory problems or dementia, carers of those with memory problems or dementia, and healthy volunteers, to register their interest in taking part in research (https://www.joindementiaresearch.nihr.ac.uk/). When recruiting through local support groups, staff at the group made the initial contact with the participant, to determine if they were interested in taking part in this study. When the participant expressed an interest, their contact details were passed on to the researcher. The researcher then reached out to the potential participant via telephone or email, depending on the preference of the participant, to explain the purpose of the study and if the participant was interested in taking part, arrange a time and date to complete the interview. Once the interview was scheduled, the researcher sent the participant a copy of the consent form to read before the interview. The onset of the COVID-19 pandemic during data collection limited our available recruitment sources and our ability to interview a larger sample of participants.

### Sampling strategy

We used purposively sampled participants based on the time since diagnosis to explore a diversity of perspectives. Participants were not limited to those who have been newly diagnosed as we wanted to explore the long-term and short-term benefits of an early diagnosis. For this study, an early diagnosis was defined as a diagnosis close to the onset of symptoms (as reported by the participant) or a diagnosis of MCI. We included participants caring for people with dementia with or without an early diagnosis.

Participants held characteristics that were highly specific to the study and provided rich and relevant data. For example, three participants were caring for two or more relatives with dementia concurrently and were able to reflect on how an early diagnosis may or may not be beneficial in different contexts. Data collection and analysis were completed in parallel with recruitment continuing until the sample held sufficient information power [20] and the analysis was considered to have achieved conceptual depth.

### Data collection procedures

All interviews were conducted by the first author, a female Ph.D. student who had previously worked as an assistant psychologist providing support to caregivers and was experienced in conducting interviews in research settings. The interviewer did not have a relationship with the participants before commencing the research. The interviews lasted for 45 min on average (range = 25–75 min) and were conducted between January and December 2020. The COVID-19 pandemic affected the data collection procedures for this study. Before the pandemic, participants had the option of an in-person interview either in their own home or at King’s College London. One interview was conducted face-to-face, in the participant’s home, before the start of the pandemic. During the pandemic, all interviews were conducted virtually using Microsoft Teams or over the phone, depending on the preference of the participant.

At the start of the interview, the researcher introduced herself, described the aims of her thesis, and explained the purpose of the study. She then asked for permission to start the recording to complete the consent form with the participant over the phone. Once the consent form had been completed, the researcher asked for permission to continue to the interview. The semi-structured interviews followed a topic guide (see supplementary file 1) and started with questions about how and when they started to notice the person with dementia’s memory problems. This was followed by questions about their experiences and the timeliness of the diagnosis and subsequent post-diagnostic support. The topic guide was initially developed in consultation with the South London and Maudsley NHS MALADY public and patient involvement group and was revised iteratively to follow the concerns of the participants.

### Data analysis

The audio recordings of the interviews were transcribed verbatim. Five recordings were transcribed by the first author and 9 were transcribed by a professional service. All transcripts were checked for accuracy and then were uploaded to NVivo 2020 for analysis. The interviews were analysed following Braun and Clarke’s six steps for reflexive thematic analysis [21, 22]. An initial list of codes was developed by three authors (EC, MC, and CA), who independently read one interview, and each identified a list of inductive codes. The three lists of semantic and latent codes were compared during a meeting in which the team talked through different interpretations of the data. A single list of codes was developed and the lead author applied this to the data. A line-by-line approach was taken to ensure all parts of the data were given equal consideration [23]. From this initial coding, the lead author reviewed the data contained in the codes to identify patterns of meaning, which were drawn out into themes. During this process, the lead and senior author meet weekly to discuss analytical decisions and the development of the themes. Contradictory data were drawn out and included in the analysis to ensure credibility [24]. Quotes from participants are presented with pseudonyms.

## Results

### Participants

We conducted interviews with 12 caregivers of people with dementia or MCI (see Table 1). Most participants were female (75%), with a mean age of 61, married or living with a current partner (75%), and a current caregiver (83%). Four participants cared for their spouse, four were caring for a parent and four were caring for more than one person with dementia. Of the four participants caring for multiple people with dementia, three were caring for both parents concurrently and one participant was caring for four close relatives (the exact relationships are not reported to maintain participant anonymity). Ten of the 12 participants were caring for someone diagnosed with dementia and eight participants were caring for someone who had received an early diagnosis of dementia. Two interviewees were former caregivers.

**Table 1:** Participant characteristics

Characteristic	*N* = 12
Gender (%)	
Female	9 (75)
Male	3 (25)
Mean Age (SD)	61 (12.5)
Marital Status (%)	
Married or Cohabitating	9 (75)
Divorced, Widowed or Currently Single	3 (25)
Current caregiver (%)	
Yes	10 (83)
No	2 (17)
Relationship to person with dementia (%)	
Spouse	4 (33)
Child of one parent with dementia	4 (33)
Caregiver to multiple people with dementia	4 (33)
Type of diagnosis (%)	
Mild cognitive impairment	2 (17)
Dementia	10 (83)
Early diagnosis (%)	
Yes	4 (33.3)
No	8 (66.7)

In this paper, we present a combination of experienced and hypothetical benefits of an early diagnosis. Participants in this study described how an early diagnosis could help them both manage the day-to-day care for the person with dementia and plan for the future. We identified three benefits of an early diagnosis: (1) protecting the person with dementia from financial or physical harm, (2) timely decision-making, and (3) access to services.

**Fig. 1:**
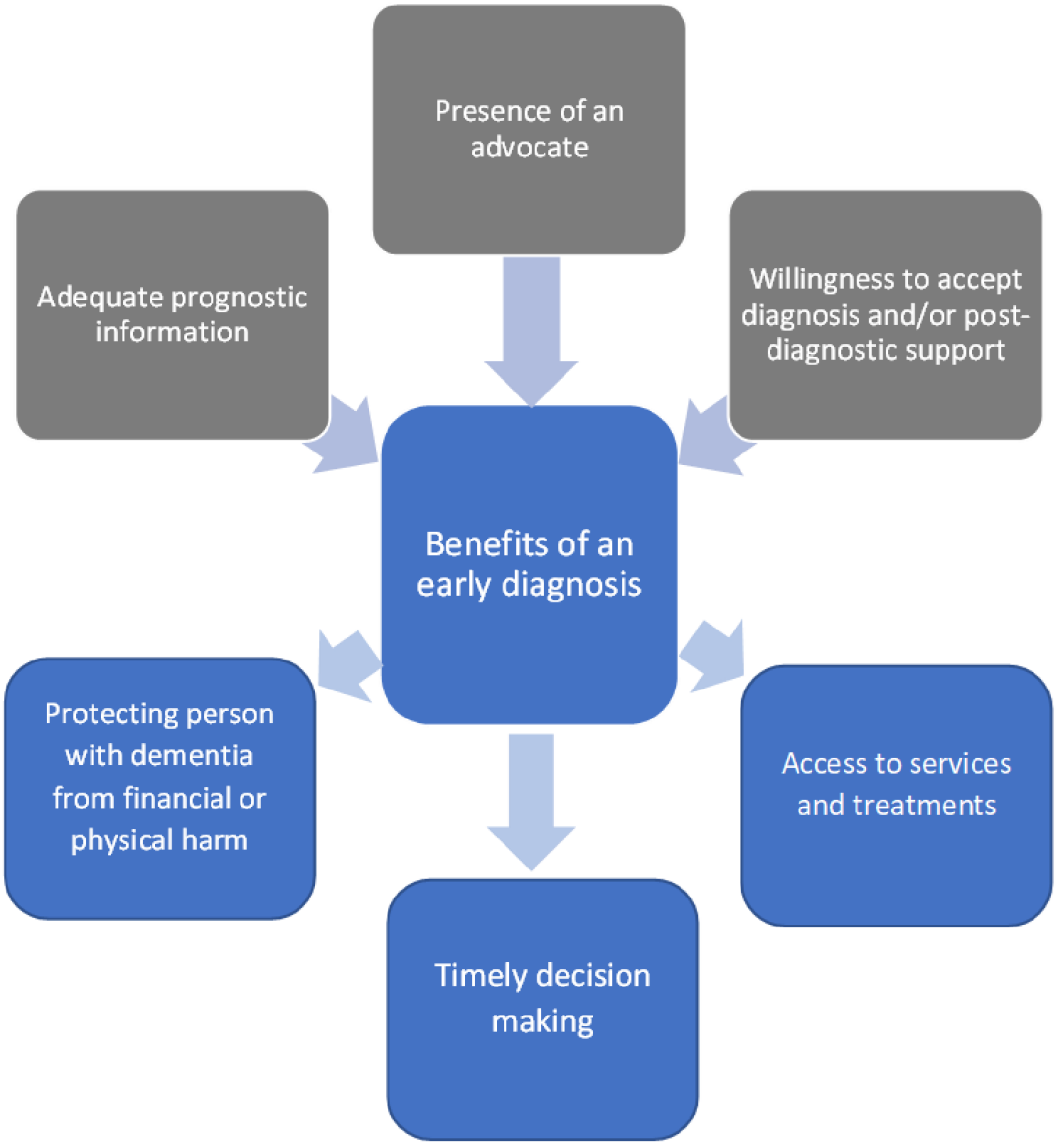
Conceptual diagram of the benefits of an early diagnosis

We also identified three conditions for an early diagnosis to be beneficial: (1) adequate prognostic information, (2) a willingness to accept the diagnosis, and (3) someone to advocate on behalf of the person with dementia. Figure 1 presents a conceptual diagram of the themes discussed in this paper.

### Benefits of an early diagnosis

Participants described how learning the person they care for has dementia could enable them to better organise the person with dementia’s care and prepare for their caregiving role. More specifically, an early diagnosis could protect the person with dementia from financial or physical harm, enable timely decision-making between the caregiver and the person with dementia, and enable early access to services.

### Benefit 1: protecting the person with dementia from financial or physical harm

Participants described how the person with dementia’s symptoms gradually developed from mild changes in memory and behaviour to more substantial cognitive impairment. Some participants said they suspected these changes in cognition were due to dementia, whereas others attributed these changes to normal ageing. During the early stages of memory loss, participants described how many of the people they became caregivers for were managing their finances and/or driving. Some participants reported that before the person with dementia was diagnosed, they were victim of financial exploitation, or scams, or were at risk of a road traffic accident. In each of these cases, participants did not feel they were able to intervene in the person with dementia’s finances or decision to continue driving until after a formal diagnosis was made.

For example, after a formal diagnosis, one participant learned the person with dementia had previously been a victim of financial scams:

When I first started investigating her bank account, she was spending £2000 a year on various home insurances, nuisance call stopping services. (Elizabeth, caregiver to a person with MCI, early diagnosis).

Similarly, another participant who reported concerns that her mother had been financially exploited by another family member, noted she was unable to access her mother’s bank account until she had provided proof of a dementia diagnosis:

Because there was one account where mum was going seriously overdrawn because there was some policy that was still being paid out of it. I had to really convince [the bank] really to let me help… And I proved that I was her daughter and that she had dementia and all of this and they let me sort it out (Rebecca, caregiver to a person with dementia, not an early diagnosis).

Similarly, participants outlined how a formal diagnosis of dementia triggered discussions or concerns regarding whether the person with dementia was still able to drive safely. For example, one participant described how she had previously been concerned about her husband’s safety while driving and how she persuaded him to stop after his diagnosis and a near miss:

He was borderline driving, but certainly soon after [the dementia diagnosis], after he’d driven through a red light and things, I persuaded him he’d got to stop driving. (Sarah, caregiver to a person with dementia, not an early diagnosis).

Another participant, who also had expertise in transport, echoed the importance of a driving test triggered by the dementia diagnosis:

And I can’t tell you how many times I’ve been there, watched an elderly person have a diagnosis test, and the person be told ‘Well I’m sorry but you really can’t have your driving licence renewed’, and behind them, the family’s going ‘yes!’ Because finally, they’ve got the driving licence off Dad rather before he kills someone. (Catherine, caregiver to multiple people with dementia, not early diagnosis).

### Benefit 2: timely decision making

Participants described how receiving a diagnosis prompted them and the person with dementia to make plans for their future care. Most participants said they went through the legal process to assign power of attorney immediately after the diagnosis:

But as soon as we found out he’d got dementia, we started thinking about solicitors. We’ve done a power of attorney. We’ve done the will. (Sheila, caregiver to a person with dementia, early diagnosis).

They highlighted how an early diagnosis could allow people with dementia to assign power of attorney to the person they believe will make decisions with their best interests at heart:

“Me and my brother, at an earlier stage in my dad’s dementia were, we got power of attorney so that we could make decisions for his care if we needed to… me and my brother were the oldest and we did it like that so that we could both… He would have some balance.” (Joanne, caregiver to a person with dementia, early diagnosis).

Caregivers found this process to be so important that many said they had arranged for their own power of attorney following their experience of caregiving:

It was about that time that [my brother and I] thought we should take out power of attorney for each other. So, we got that started. (Carol, caregiver to multiple people with dementia, not an early diagnosis).

Participants reported an awareness that the person with dementia’s symptoms would progress to a point where formal care would eventually be needed. They described how an early diagnosis could trigger conversations with the person with dementia about their care preferences. Participants noted that knowing the person with dementia’s preferences for future care could bring comfort when making difficult decisions. For example, one participant, caring for his wife with an early diagnosis, described having a “game plan for the long-term” which he had discussed with his wife following her diagnosis. On the other hand, another caregiver to her husband with a later diagnosis described finding the prospect of introducing formal care without having previously discussed her husband’s wishes challenging:

We never had the care home conversation. Not seriously. My view is that if I get old and craggy I want to be in a care home and not be a burden to my family. But I’m not sure he feels the same way, so it’s a bit difficult for us now, not knowing where he stands (Sarah, caregiver to a person diagnosed with dementia, not an early diagnosis).

### Benefit 3: Access to services

Participants described how accessing services early, as a result of an early diagnosis, could help them better prepare for their future caregiving role. Participants reported that the formal diagnosis of dementia enabled the person with dementia to access specialist services and treatments, including both pharmacological and non-pharmacological treatments:

She has had quite a lot of support including going to some kind of classes to help for people with dementia and taking medication too (Catherine, caregiver to multiple people with dementia, not early diagnosis).

In the early stages of the disease, participants were most interested in the person with dementia receiving anti-dementia medications to slow down the symptoms of cognitive decline. Some participants reported an awareness that anti-dementia medications were likely to be more effective if delivered early. The desire for early access to services and treatments motivated some participants to prompt the person with dementia to seek a formal diagnosis in the first place.

Participants also described how services could provide information on how to best support the person with dementia. They were most interested in knowing what symptoms or behaviours were likely to arise next coupled with practical information on how to treat and manage these symptoms:

What does this mean on a day-to-day basis? And how can I help mum through this? So, I guess that’s more my focus on the symptoms, and what to do to help. (Rebecca, caregiver to a person with dementia, not an early diagnosis).

Participants reported a preference for this information and support as early as possible. For example, one participant described how she wanted earlier support for managing her Dad’s difficulties with eating:

So yeah, so lots of the physical things, we could have done with a lot more guidance about, and advice on equipment earlier on, rather than having to see my dad just starve, because he couldn’t eat anything until we worked out the best way of preparing his food. (Joanne, caregiver to a person with dementia, early diagnosis).

### Conditions for an early diagnosis to be beneficial

Participants highlighted weaknesses in the current provision of dementia care that might prevent the benefits of an early diagnosis from being felt. Overall, we identified three conditions needed to enable the benefits of an early diagnosis: adequate prognostic information, a willingness to accept the diagnosis, and someone to advocate on behalf of the person with dementia.

### Condition 1: adequate prognostic information

As discussed previously, participants described how an early diagnosis could help manage the day-to-day care for the person with dementia and make plans for the future. However, participants noted that this could only be possible with personalised and timely prognostic information. Participants reported difficulties in finding such information and described how this could be offered by health services.

If we could have some kind of community-based assessment, [with] somebody with expertise who could spend some time [with us] in order to be able to say, Oh yes, I can see what’s going on here. I can see how this is going to go. This is what we can do about it. Or there is nothing we can do about it, and I’m afraid inevitably, this is what’s going to happen very soon. Or something like that. You know, that’s what I wanted. (James, caregiver to multiple people with dementia, not an early diagnosis).

Additionally, participants highlighted the need for a single source of information for finding individualised advice. They described going to books, support groups, dementia or ageing charities, health and social services, friends and family, newspapers, online videos, and doing independent research on Google. Often, they did not feel the information available was relevant to their circumstances:

I guess every situation is different and I haven’t sort of found anybody who I can say, well, that’s just like me and my situation. (Mark, caregiver to a person diagnosed with dementia, early diagnosis).

When describing the ideal dementia service, participants described a one-stop shop offering advice for both the person with dementia and their caregiver. Participants said they wanted regular follow-ups, especially immediately after diagnosis, as this would enable access to individualised information at the right time. They felt that this was important for managing the emotional and practical impact of a diagnosis, especially in the weeks and months following the diagnosis:

I think it would be really good if there had been someone who I could have spoken to maybe once a month or more, maybe once every two months maybe. (Sarah, caregiver to a person with dementia, not an early diagnosis).

Even where participants reported receiving insufficient information and support from health services, they described how an early diagnosis could help them to better understand the person with dementia:

Interviewer: What I’m hearing is it doesn’t make much difference whether you’re diagnosed early or late. There’s… not much for you.

Participant: I think that’s, I would say that’s correct, yes, but it might make a difference to how you get on with the person, actually…It is an important consideration because very often it’s going to be the partner or whoever, or a close family member who’s going to be the prime carer. Since there is so little support. So, if they know sooner rather than later that this is a disease and not their loved one being [difficult]. Then it’s going to be a bit helpful… won’t necessarily be easier… (Sarah, Sarah, caregiver to a person with dementia, not an early diagnosis).

And to prepare for the emotional impact of caring for someone with dementia:

But just the anguish of the long goodbye. I think maybe an earlier diagnosis would certainly help you prepare, prepare more, I think. Although it doesn’t take the pain away from the length of time. You know. That is something you just have to bear with. (Rebecca, caregiver to a person with dementia, not an early diagnosis).

### Condition 2: Presence of an advocate

For many participants, the availability of someone to advocate on behalf of the person with dementia was perceived as vital for ensuring a good quality of life as the person with dementia’s cognitive impairment progressed. While an early diagnosis could enable people with dementia to express their preferences for their future care, participants described how, as caregivers, they would be the ones to ensure the person with dementia’s wishes were acted on.

We made these plans after his diagnosis, but quite a few years ago now, actually. And we both sort of said to my dad ‘We will do our absolute best to keep you in the home for as long as possible’. (Joanne, caregiver to a person with dementia, early diagnosis).

Caregivers described how they often acted as a broker between services and the person with dementia, facilitating access to specialist services. Participants felt that the person only got the amount of care they did because they “fought” for it from services. When asked to reflect on what they would do if they suspected they had dementia, most participants said they would seek a diagnosis when they noticed symptoms. For participants without someone to act as an advocate, an early diagnosis could be especially important for making early plans to ensure they get the care they need during the later stages of dementia:

I would make sure that I got checked. I would probably start to put some things in place…I don’t think children or marriage are an insurance policy anyway. But you become very aware of the fact that if you start to have Alzheimer’s, it’s going to be really, really difficult and that you will need to put things in place before you’ve completely lost the ability to communicate or fight for your rights or feed yourself, all those sorts of things. (Joanne, caregiver to a person with dementia, early diagnosis).

Conversely, other participants without potential future advocates described fears they would be vulnerable to receiving poor care. Such concerns were so overwhelming, that participants reported potentially wanting to end their lives early:

I won’t have anyone who will… be my advocate like I was with my parents. You know I did all their finances and everything. I just think it would be a nightmare. Beachy Head or something. I don’t know if I can’t find a pill. (Carol, caregiver to multiple people with dementia, not an early diagnosis).

And:

Participant: Hmm. I think you probably could live with it longer… You know. If you had that option to be… To have your life ended, once you get to a certain stage, then you would…It would be worth living to that stage….

Interviewer: Having that option set up. It sounds like what you’re describing is a sort of freedom, I guess, or relief?

Participant: Yes. Yes, it is. Yes. Yes, it would be very much that… It would be a terrific relief to have that there as an option in the future. (Elizabeth, caregiver to a person with MCI, early diagnosis).

### Condition 3: willingness to seek and accept the dementia diagnosis

Participants noted how a person with dementia’s refusal to seek or accept a diagnosis could be a barrier to an early diagnosis and its previously described benefits. There was variation in whether the person with dementia or the caregiver initiated the process of seeking a dementia diagnosis. One participant noted that his wife, with dementia, had initiated the diagnostic process in the early stages of dementia, which enabled them to organise support:

She noticed her memory issues if she couldn’t remember things… So she started the ball rolling, but I think after a while, I, obviously, got very concerned and tried to get as much support and help as we could. (Mark, caregiver to a person with dementia, early diagnosis).

However, most participants in this study reported that they had noticed the person with dementia was experiencing problems with their memory and encouraged them to get tested for possible dementia. In many cases, the person with dementia was reluctant to seek a diagnosis for their memory problems:

I definitely had that conversation and said, ‘I know it’s scary. But there’s something wrong and you need to face it.’ But she just looked at me blankly and refused to engage. (Rebecca, caregiver to a person with dementia, not an early diagnosis).

Even after the formal diagnosis was given, some participants noted the person with dementia did not accept their diagnosis:

There was probably a period of about six more months, I would say, he didn’t want to go to the GP to talk about it. He never in this life acknowledged that he had dementia. (Sarah, caregiver to a person with dementia, not an early diagnosis).

And:

He knew there was some problem, but he wouldn’t. He didn’t really want to accept what, or what implications were, I’d say. (Carol, caregiver to multiple people with dementia, not early diagnosis).

Some participants linked the unwillingness to seek a diagnosis or the lack of acceptance once the diagnosis was given to delays in access to services and planning for the future:

The biggest obstacle we’ve had was him spending four years not acknowledging the situation he was in. That was the most stressful time, and it wasn’t because he didn’t get the support, it was because he wouldn’t accept that support. (Catherine, caregiver to multiple people diagnosed with dementia, not early diagnosis).

## Discussion

In this study, we identified three benefits of an early diagnosis of dementia from the perspective of caregivers. An early diagnosis could (1) protect the person with dementia from harm during the early stages of memory loss, (2) enable timely decision making and (3) enable access to services and treatments. We also identified three conditions necessary for an early diagnosis to be beneficial (1) adequate prognostic information, (2) someone to advocate on behalf of the person with dementia and (3) the person with dementia being willing to seek and accept a diagnosis.

Participants described how an early diagnosis of dementia could enable caregivers to intervene during the early stages of cognitive impairment to protect the person with dementia from harm. Participants particularly noted this was important for protecting people with dementia from financial harm or a road traffic accident. This is supported by previous research which found that difficulties in managing finances is one of the earlier symptoms of dementia and assessment of financial capacity can identify people with MCI who are most likely to progress to dementia [25, 26]. Financial abuse is the most prevalent form of elder abuse in the UK [27] and people with MCI or dementia are at greatest risk [28]. Fenton et al. suggest that the early diagnosis of dementia can help identify those most at risk of financial exploitation, empower such individuals to avoid exploitation, and reduce the societal costs and consequences of exploitation [29]. Similar arguments can be made regarding an early diagnosis reducing the risk of road traffic accidents among people with MCI or dementia. People with MCI and dementia showed impaired driving performance compared with cognitively healthy individuals [30]. Despite this impairment people with MCI may be able to drive safely for many years following the onset of symptoms [30]. An early diagnosis can make people with dementia, their caregivers, and clinicians aware that the persons with driving ability will need to be regularly assessed and help them to identify an appropriate time for them to cease driving [30, 31].

As their cognitive impairment worsens, people with dementia and MCI increasingly rely on caregivers or proxies for decision-making. Participants in this study described how an early diagnosis could enable caregivers to make decisions and plans for the future together. Previous research indicates that caregivers value the opportunity to make practical arrangements. Vernooij-Dassen et al. found that within three months of diagnosis, some people with dementia and their caregivers reported making significant life decisions, including getting married or moving house [32]. An early diagnosis enables people with dementia to be more involved in such decision-making. Participants also noted that knowing the person with dementia’s future care preferences could bring comfort during the later stages of the disease. However, people with dementia may feel differently about making decisions following a diagnosis of dementia. Some participants in this study reported that the person with dementia was reluctant to accept the diagnosis and engage in decision-making, treatments, or support, which may present a barrier to the benefits of an early diagnosis. People with dementia may be less willing to seek or accept a diagnosis due to fear of stigma [33]. Furthermore, being provided a diagnosis before they are ready to process it could be harmful to the person with dementia’s well-being [34, 35]. Future research must examine the value of an early diagnosis from the perspective of the person with dementia.

Relatedly, participants highlighted the importance of having someone to advocate on behalf of the person with dementia. They reported concerns that people with dementia without advocates were at risk of poorer care. However, it is important to note that this sample only consisted of caregivers who may therefore have a heightened awareness of the role of caregivers as advocates. Previous research does suggest that the availability of a family caregiver may be associated with better quality care. For example, one study in the US found frequent visits from family members during a nursing home stay were associated with better quality care [36]. It is estimated that 25% of people with dementia are not supported by informal caregivers, meaning there is a significant proportion potentially lacking important support [13]. The effects of caregiving are well documented. People with dementia who are living alone with little support report less satisfaction with life and higher levels of loneliness [37] however, more research is needed to better understand the needs and experiences of this population. All people with dementia should equally benefit from a diagnosis, early or otherwise, therefore future research should examine whether outcomes differ between people with dementia who have support from a caregiver and those who do not.

Early access to services and support was identified by participants as a key benefit to an early diagnosis. They described how people with dementia were able to access nonpharmacological and pharmacological treatments to manage their cognitive decline, however, participants were aware that such treatment may be more effective if delivered during the early stages of dementia. There is evidence to suggest that early engagement with community and respite services could delay institutionalisation for people with dementia [17]. However, participants also described struggles with accessing and organising post-diagnostic support from health and social care services. On the one hand, it could be argued that ineffective post-diagnostic support could present a barrier to an early diagnosis being beneficial. On the other hand, an early diagnosis could provide people with dementia and their caregivers with extra time to navigate the complexity of dementia services. Either way, effective and well-organised post-diagnostic support is critical for improving the health and well-being of people with a diagnosis of dementia and their caregivers, early or otherwise [38]. Participants in this study highlighted the need for a single service that could provide individualised prognostic information over the course of the disease. They specifically wanted earlier information on what symptoms to expect, how to manage them when they arise, and an annual review. These findings are similar to other studies investigating priorities for post-diagnostic support following a dementia diagnosis [39, 40] and lend further support to efforts to design more responsive services [41, 42]. Even in cases, where post-diagnostic support was perceived to be inadequate, participants described how the early knowledge that the person with dementia’s symptoms are caused by dementia can help them to better understand that person and prepare for their future caregiving role. Previous research has indicated that this preparation for a future caregiving role can be particularly important for caregivers to people diagnosed with MCI [43].

### Strengths and limitations

This study provides valuable information on caregiver perspectives on the benefits of an early diagnosis. However, the findings should be interpreted in the context of this study. This study was part of a larger Ph.D. project and the COVID-19 pandemic started during data collection for this study. The pandemic and subsequent restrictions on time and funding limited our ability to recruit a larger sample of participants for this study. Participants were identified through an online dementia registry and local support groups, meaning they were likely to be well-engaged with existing support and dementia services. Caregivers who are not as engaged with existing services may have differing perspectives, which warrants future investigation. Participants with an early diagnosis were identified via self-report, future research could consider using electronic health care records or structured cognitive assessments of the person with dementia to identify caregivers to people with an early diagnosis. Furthermore, participants in this study were largely white and middle-class. Crucially, only caregivers were interviewed in this study, therefore future research should examine the perspectives of people with dementia. Although this study reports a small and relatively homogeneous sample, the findings provide valuable insight into an important issue in dementia research and practice. Future research is needed to expand on this study with a larger and more diverse sample.

## Conclusions

The findings of this study lend empirical support for three benefits of an early diagnosis: protecting the person with dementia from harm during the early stages of the disease, timely decision-making, and access to services and treatments following a diagnosis. However, we also identified three conditions necessary for the benefits of an early diagnosis: adequate prognostic information, a willingness to accept the diagnosis, and someone to advocate on behalf of the person with dementia. Overall, an early diagnosis can enable people with dementia and their caregivers to make practical arrangements for the person with dementia’s current and future care. Further research is needed to expand on the findings of this study with a larger, more diverse sample and the inclusion of people with dementia.

### Electronic supplementary material

Below is the link to the electronic supplementary material.

Supplementary Material 1

## Data Availability

The datasets generated and/or analysed during the current study are not publicly available due to the sensitive and personal nature of this topic. A limited dataset is available from the corresponding author upon reasonable request.

## Abbreviations

JDR: Join Dementia Research
NHS: National Health Service
MCI: Mild cognitive impairment
