# Supplementary material for: A qualitative study of informal caregiver perceptions of the benefits of an early dementia diagnosis
Source: BMC Health Serv Res. 2024 Apr 24;24:508. doi: 10.1186/s12913-024-10957-6 (PMC11040854; doi:10.1186/s12913-024-10957-6)
Supplement: Supplementary file 1 — Supplementary Material 1 [file 12913_2024_10957_MOESM1_ESM.docx]

**Identifying the benefits of early diagnosis in dementia**

**Topic guide for carers**

**Experiences of the person living with dementia receiving the diagnosis**

Tell me about when you first started to notice problems with [name of PLwD]’s memory?

What did you initially attribute [PLwD]’s to? What did you think was causing their memory problems?

Did you speak to anyone about [PLwD’s] memory problems?

When did you decide to seek help for [name of PLwD’s] memory problems?

Would you usually go to the doctors?

Can you tell me about the experience of getting the diagnosis of dementia? Prompt on:

- Expectations of diagnosis (e.g. what did you expect to happen? Were those expectations met? Hopes? Worries?)
- What was the process of getting a diagnosis? E.g. memory tests
- What was the impact of the diagnosis on you (e.g. emotional, practical etc.)
- What was impact on [PLwD]? (e.g. emotional, practical, etc.)
- What was helpful/unhelpful in this experience?

Reflecting on your experience of diagnosis, are there any ways in which you think finding out about [PLwD]’s diagnosis sooner would have helped, or do you feel you found out at the right time? Why?

What did the diagnosis mean for [PLwD]?

**Experiences of post-diagnostic support**

When you went for the diagnosis, did you expect [PLwD] to receive any treatment (e.g. drug treatment, cognitive therapies, group support)?

Did you or [PLwD] receive any treatment or support?

Prompt on:

- Common dementia drug treatments
- Cognitive stimulation therapy
- Occupational therapy
- Support groups
- Invitations to take part in research
- Carer’s assessment
- Carer’s support group
- Local authority services

If yes, can you tell me a little bit more. What was your experience of receiving this treatment/support? What was helpful or unhelpful.

Did you stop using this treatment/support? If yes, why?

Did you access any other forms of support? (e.g. church) Why? Can you describe what type of support you received and how this was helpful or unhelpful?

What support do you think is needed for PLwD or cargivers, especially in the early stages of dementia?

Is there any support that you didn’t have but wish you did? If yes, what was it and how would this have helped?

Did the diagnosis change how you think or feel about memory problems?

**Experiences of Health Services**

Have you used any health services including:

- GP
- A&E
- hospital stays
- care homes

If yes, tell me about that experience? Why did you use this service? What was helpful or unhelpful?

**Planning for the future**

- Have you or [PLwD] made any plans for their future?
- If yes, can you tell me about them?
- How has [PLwD]’s diagnosis affected these plans? Did your plans change as they disease has been progressing?

**Concluding questions**

Thank you for answering my questions, is there anything else you would like to tell me about your experiences which I haven’t asked about?
